# Pulmonary Vein Anatomy is Associated with Cryo Kinetics during Cryoballoon Ablation for Atrial Fibrillation

**DOI:** 10.5935/abc.20180071

**Published:** 2018-05

**Authors:** Xiongbiao Chen, Pihua Fang, Zheng Liu, Jia He, Min Tang, Jun Liu, Bin Lu, Shu Zhang

**Affiliations:** 1 Department of Cardiac Arrhythmia - State Key Laboratory of Cardiovascular Disease - Fuwai Hospital - National Center for Cardiovascular Diseases - Chinese Academy of Medical Sciences and Peking Union Medical College, Beijing - China; 2 Department of Radiology - State Key Laboratory of Cardiovascular Disease - Fuwai Hospital - National Center for Cardiovascular Disease - Chinese Academy of Medical Sciences and Peking Union Medical College, Beijing - China

**Keywords:** Pulmonary Veins / anatomy & histology, Atrial Fibrillation, Catheter Ablation, Multidetector Computed Tomography, Cost-Benefit Analysis

## Abstract

**Background:**

The influence of pulmonary vein (PV) anatomy on cryo kinetics during cryoballoon (CB) ablation is unclear.

**Objective:**

To investigate the relationship between PV anatomy and cryo kinetics during CB ablation for atrial fibrillation (AF).

**Methods:**

Sixty consecutive patients were enrolled. PV anatomy, including ostial diameters (long, short and corrected), ratio between short and long diameters, ostium shape (round, oval, triangular, and narrow), and drainage pattern (typical, with common trunk, common antrum, ostial branch and supernumerary PV) were evaluated on multi-detector computed tomography (MDCT) images pre-procedure. Cryo kinetics parameters [balloon freeze time from 0 to -30ºC (BFT), balloon nadir temperature (BNT) and balloon warming time from -30 to +15ºC (BWT)] were recorded during procedure. All p values are two-sided, with values of p < 0.05 considered to be statistically significant.

**Results:**

606 times of freezing cycle were accomplished. Moderate negative correlation was documented between BNT and corrected PV diameter (r = -0.51, p < 0.001) when using 23-mm CBs, and mild negative correlation (r = - 0.32, p = 0.001) was found when using 28-mm CBs. Multivariate logistic regression analysis revealed that PV corrected ostial diameter (OR, 1.4; p = 0.004) predicted a BNT < -51ºC when using 23-mm CBs, while PV ostium oval shape (OR, 0.3; p = 0.033) and PV locations (left inferior PV: OR, 0.04; p = 0.005; right superior PV: OR, 4.3; p = 0.025) predicted BNT < -51ºC when using 28-mm CBs.

**Conclusions:**

MDCT can provide PV anatomy accurate evaluation prior CB ablation. PV anatomy is associated with cryo kinetics during ablation.

## Introduction

CB ablation has an increasing clinical application worldwide, it has been proved a comparable technique to radiofrequency (RF) ablation in safety and efficacy for the AF treatment,^1^ and maybe more cost-effective.^2^ By achieving appropriate occlusion in targeted PVs with the balloon and getting good balloon - PV ostium contact, it can simplify the procedure with a “single-shot” approach to get circumferential PV isolation.^3^ It is reported that some parameters of cryo kinetics, such as balloon temperature,^4^ balloon warming time,^5^ can predict acute PV isolation or late PVs reconnection. Some parameters of PV anatomy have been used to predict occlusion,^6^ or acute, mid- and long-term success of CB ablation.^7^^-^^9^ It is reasonable to imaging that PV anatomy plays a role in cryo kinetics, thus exerting an influence on ablation efficacy. However, limited data exist regarding the association between PV anatomy and cryo kinetics during CB ablation. We aimed to investigate the relationship between PV anatomy parameters and cryo kinetic parameters in patients undergoing CB ablation using either 23- or 28-mm CB for AF.

## Methods

### Patients

Between January and October 2014, a prospective study was carried out at our institution. Sixty consecutive patients with symptomatic and drug-refractory AF underwent CB ablation. In these patients, pre-procedural MDCT images and complete recordings of cryoballoon temperature during each CB ablation were available. All patients provided written informed consent. The study followed the ethical standards of the Declaration of Helsinki of 1975, revised in 2008 and was approved by the local institutional ethics committee.

### PV Anatomy Assessment

#### Image acquisition

Prior to the procedure, MDCT studies were performed on a MDCT scanner (SOMATOM Definition Flash, Siemens). Scanning parameters were the following: tube voltage 100 - 120 kV, 3D automatic tube current modulation, thickness / increment of reconstruction 0.625 / 0.625 mm. ECG-gating was not used, and patient breath holding was required during image acquisition. A bolus tracking protocol with 50 ~ 70 mL i.v. contrast agent (Ultravist 370, Bayer Schering) and 3 ~ 5 mL/s flow rate was applied.

### Image analysis

**Figure 1:**
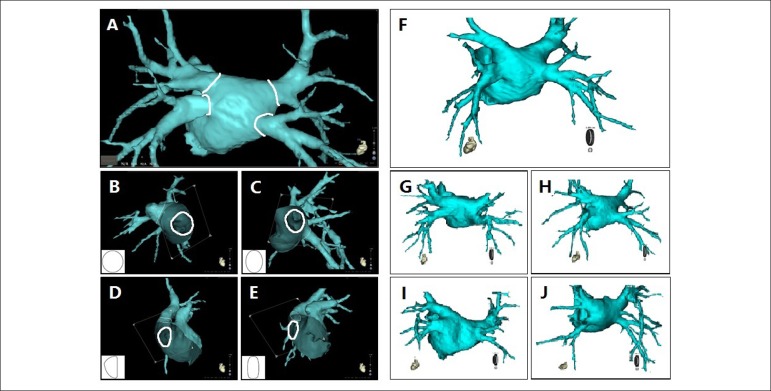
PV ostium shapes category and PV drainage patterns. A) PV ostia depicted semi-automatically using CartoMerge software. Four shapes of PV ostium; B) Type I (round); C) Type II (oval); D) Type III (triangular); and E) Type IV (narrow). Five patterns of PV drainage; F) Four PVs drains into LA in a typical pattern; G) Left superior and inferior PVs drains into LA both in a pattern of with common trunk; H) Left superior and inferior PVs drains into LA both in a pattern of with common antrum; I) Right inferior PV drains into LA in a pattern of with ostial branch; J) Right superior and inferior PVs drains in LA with supernumerary vein (middle vein).

MDCT images were reconstructed and analyzed using CartoMerge software (Biosense Webster, Diamond Bar, CA, USA) right before the procedure. PV ostia were defined anatomically at the parietal pericardium point of reflection^10^ and were depicted semi-automatically (Figure 1A), together with ostia perimeters calculated automatically by computerized image analysis. Long (D_long_) and short (D_short_) ostia diameters were then measured. Corrected ostial diameters (D_corrected_) were calculated using the formula D_corrected_ = perimeter / π. The ratio between D_short_ and D_long_ (D_short_ / D_long_) was also calculated for analysis. Taking consideration of D_short_ / D_long_ values, PV ostium shapes were divided into 4 types: type I (round), ostia with value between 0.90 ~ 1.00; type II (oval), value between 0.60 ~ 0.90 and a smoothly curved edge; type III (triangular), value between 0.60 ~ 0.90 and an obviously straight part at the edge; and type IV (narrow), value less than 0.60. (Figure 1B-E).

Five PV drainage patterns were defined for the targeted superior/inferior PVs based on the definition by Marom et al.^11^ When the superior and inferior PVs on the same side joined together to form a common trunk vein and drained into LA through a common ostium, the superior and inferior PV were defined as “with common trunk”. If the superior and inferior PVs on the same side drained into LA through two independently trunk but drained into LA through ostia hardly separated by LA wall (the minimum distance between the two ostia was less than 2 mm on MDCT images), the two PVs were then defined as “with common antrum”. PV “with ostial branch” was defined as a PV branch joining within 10 mm from the ostium. PV “with supernumerary vein” was defined as the superior or inferior PV with neighboring additional vein(s), when a middle PV existed, both the superior and inferior PV on the same side were defined as “with supernumerary vein”. PV “with typical drainage” was defined as a superior or inferior PV drained into LA independently, through neither a common trunk nor antrum, and that did not have an ostial branch or supernumerary vein. (Figure 1F-J)

### Anatomical assessment reproducibility

In order to assess evaluating methods reproducibility of diameters described above, 40 PVs ostial diameter of first 10 patients were measured on CT images by two blind experienced observers at the beginning of the study. One observer measured two times in different moments to study the inter-observer reproducibility. The other observer measured one time, and the intra-observer reproducibility between the two observers was studied. The ostium shapes and drainage patterns were also assessed by two experienced observers in consensus during the study.

### Ablation procedure

The ablation procedures were carried out as previously reported.^12^ Briefly, an octapolar electrode catheter was placed into the coronary sinus and a phrenic nerve (PN) pacing electrode catheter into the superior vein cava (SVC). After a single transseptal puncture, selective PV angiography was carried out and a CB catheter (Arctic Front, Medtronic, Quebec, Canada) was inserted into LA together with a spiral catheter (SC) (Achieve, Medtronic, CA, USA). There are currently two sizes of balloon catheters (23 or 28 mm) and two sizes of SCs (15 or 20 mm) available. PV ostia diameters were determined from MDCT images; CB and SC size were selected accordingly: If long diameters of three or four PVs were < 22 mm, 23-mm CB a 15-mm SC were selected; If that ≥ 22 mm, a 28-mm CB and a 20-mm SC were preferred; otherwise the choice would be made by the operator temporaly. As soon as good contact of balloon to PV ostium indicated by the contrast retention in PV was obtained, freezing cycle was started with two to three applications per vein. Generally each freeze lasted 240s, and ideal freezing temperature was between -45ºC and -55ºC. If exists a common PV, freezing was analyzed separately as in superior or inferior PV based on location of balloon distal end during freezing. Supernumerary PVs were not taken as targeted PV as there are usually too small in dimension.

PN was constantly paced (10 mA, 2 ms, 50/min) with PN pacing catheter in SVC when freezing at right PVs. After each freeze, PV conduction was re-evaluated by adjusting SC position within the PV. In all patients, PVI of all targeted PVs with primary use of CB only was the procedural endpoint. If PVI was not achieved for a particular vein following a minimum of two freezing, either further cryoablation would be performed or conventional RF ablation would be undertaken, depending on the initial contrast-guided occlusion and the minimum temperature achieved.

### Cryo kinetics

Three parameters of cryo kinetics^5^ were introduced: balloon freezing time from 0 to -30ºC (BFT), balloon nadir temperature (BNT) and balloon warming time from -30 to +15ºC (BWT). Freezing cycles with a BNT lower than -30ºCwere taken into analysis.

### Statistical analysis

After being tested for normality distribution and variances equality using One-Sample Kolmogorov-Smirnov test and Levene’s test, continuous variables were presented as mean ± standard deviation (SD) or median (interquartile range), and were compared using the unpaired Student’s t-test or nonparametric variables Mann-Whitney U test as appropriate. Categorical variables were expressed as number (percentage) and were compared by means of c^2^ analysis or Fisher exact test. Measuring reproducibility of PV ostial diameters was assessed using intra-class correlation coefficient (ICC). Pearson or Spearman correlation was used to evaluate the association between two variables based on its distributions. Logistic regression was performed to investigate the predictive values of PV anatomic parameters for cryo kinetic effect. Variables with a p value < 0.10 in univariate analysis were included into the multivariate analysis, which was performed using an enter approach with criteria of p < 0.05 for inclusion in and p > 0.05 for exclusion from the model. A two-sided p < 0.05 was considered statistically significant. All statistical analysis were performed using IBM SPSS statistical software (Version 20.0, SPSS).

## Results

### Study population and procedural data

The study population baseline characteristics and ablation procedure parameters are presented in Table 1.Compared with 28-mm CB only, the acute PVI rates were not significantly different when ablation was using 23-mm CB only either on PV level (92.5% vs. 96.9%, p = 0.16) or on patient level (79.4% vs. 91.7%, p = 0.28). No significant difference was found in total complication rate between 28- or 23-mm CBs (8.8% vs. 4.2%, p = 0.64). One case of PN palsy, taken as major, was detected during freezing in a right inferior PV using a 28-mm CB and did not recover until discharge. One case of pericardial and pleural effusion, two cases of left groin hematomas were all resolved within one month post-procedure.

**Table 1:** Baseline Characteristics of study population and CB PVI Procedure Parameters

Baseline Characteristics	
Age (years)	56.8 ± 12.5
Gender, male	32(53.3)
BMI (kg/m2)	24.6 ± 3.1
AF type, paroxysmal AF	58 (96.7)
AF duration (months)	25.5 (12, 69)
CHA_2_DS_2_-VASc score	1(0, 2)
LAD (mm)	35.2 ± 4.8
LVEF (%)	65.6 ± 5.4
Procedure Parameters	
Balloon type, 28-mm /23-mm/double	34 (56.7) / 24(40) / 2(3.3)
Nº. of freeze	10.8 ± 3.2
Acute PVI	
Patient level *	60(100)
With CB only, PV level^#^	225 (94.5)
With CB only, patient level	51(85)
Complications	4 (6.7)
Phrenic nerve palsy	1 (1.7)
Pericardial & pleural effusion	1 (1.7)
Left groin hematomas	2 (3.3)

Values are n (%), mean ± SD, or median (interquartile range). CB: cryoballoon; PVI: pulmonary vein isolation; PV: pulmonary vein; AF: atrial fibrillation; BMI: body mass index; LAD: left atrial diameter; LVEF: left ventricular ejection fraction (measured from transthoracic echocardiography); CHA_2_DS_2_-VASc score = stroke risk score [cardiac failure, hypertension, age ≥ 75 years (doubled), diabetes, stroke (doubled)‑vascular disease, age of 65-74 years and sex category (female)].

* PVI with CB ablation only or plus conventional RF ablation.

# 1 right superior and 1 right inferior PV has no potential

### Anatomy data

The pre-analysis on reproducibility revealed that inter-observer ICC of D_long_, D_short_ and D_corrected_ was 0.93, 0.95 and 0.96 (all p < 0.001), and intra-observer ICC of three measured diameters was 0.90, 0.96 and 0.93 respectively (all p < 0.001).

**Figure 2:**
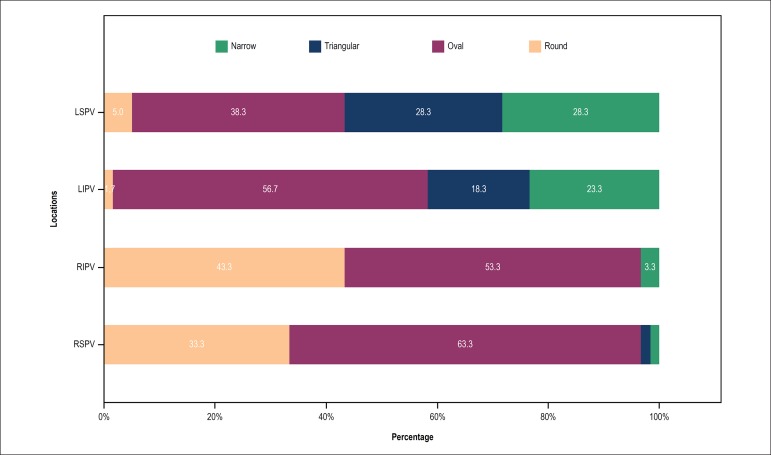
Proportions of different ostium shapes of the four targeted PVs. D_long:_ PV ostium long diameter; D_short:_ PV ostium short diameter; D_corrected:_ Corrected diameter calculated from PV ostium perimeter PV ostium; CB: cryoballoon; LSPV: left superior pulmonary vein; LIPV: left inferior pulmonary vein; RIPV: right inferior pulmonary vein; RSPV: right superior pulmonary vein.

Diameters of 240 PVs measured on CT images were listed in Table 2. Compared with ablation using 23-mm CBs, ratio of D_corrected_ and CB diameter was much smaller when frozen using 28-mm CBs (0.76 ± 0.14 vs. 0.68 ± 0.13, p < 0.001). Linear correlation analysis showed that D_corrected_ was strongly correlated with D_long_ (correlation coefficient: 0.93, p < 0.001) and D_short_ (correlation coefficient: 0.90, p < 0.001), while the latter two were moderately correlated with each other (correlation coefficient: 0.74, p < 0.001). Values of D_short_ / D_long_ were between 0.38 and 1.00. Proportions of different ostium shapes and drainage patterns of four targeted PVs are presented in Figure 2 and Table 3.

**Table 2:** PV ostia diameters measured on CT images

PV location	D_long_ (mm)			D_short_ (mm)			D_corrected_ (mm)		
	23-mm CB	28-mm CB	p	23-mm CB	28-mm CB	p	23-mm CB	28-mm CB	p
LSPV	20.3 ± 3.0	21.7 ± 2.8	0.06	13.5 ± 2.7	15.2 ± 3.3	.04	17.7 ± 2.7	19.3 ± 2.5	0.02
LIPV	17.4 ± 3.4	17.6 ± 2.1	0.80	11.7 ± 3.3	12.9 ± 2.1	.13	15.3 ± 3.2	15.9 ± 1.9	0.41
RIPV	18.3 ± 3.0	19.6 ± 3.0	0.09	15.6 ± 2.9	17.1 ± 3.0	.054	17.3 ± 2.6	18.8 ± 2.7	0.046
RSPV	21.2 ± 3.0	24.3 ± 3.4	0.001	18.0 ± 3.7	20.4 ± 3.8	.02	20.0 ± 3.0	22.7 ± 3.4	0.01
Total	19.2 ± 3.4	20.8 ± 3.8	0.001	14.6 ± 3.9	16.4 ± 4.1	.001	17.6 ± 3.3	19.1 ± 3.6	0.001

Values are mean ± SD. p: p-value (unpaired Student's t-test). D_long_: PV ostium long diameter; D_short_: PV ostium short diameter; D_corrected_: Corrected diameter calculated from PV ostium perimeter PV ostium; CB: cryoballoon; LSPV: left superior pulmonary vein; LIPV: left inferior pulmonary vein; RIPV: right inferior pulmonary vein; RSPV: right superior pulmonary vein.

**Table 3:** Proportion of PV drainage patterns

Location	typical	with common trunk	with common antrum	with ostial branch	with supernumerary vein (MPV)
LSPV	23(38.3)	11(18.3)	25(41.7)	2(3.3)	0
LIPV	23(38.3)	11(18.3)	25(41.7)	3(5.0)	0
RIPV	24(40)	0	6(10)	27(45)	4(6.7)
RSPV	37(61.7)	0	6(10)	14(23.3)	4(6.7)
Total	107(44.6)	22(9.2)	62(25.8)	46(19.2)	8(3.3)

Values are n (%). MPV: middle pulmonary vein; LSPV: left superior pulmonary vein; LSPV: left superior pulmonary vein; LIPV: left inferior pulmonary vein; RIPV: right inferior pulmonary vein; RSPV: right superior pulmonary vein.

### Cryo kinetics

238 targeted PVs were frozen 606 times. Of which, 102 PVs were frozen 254 times using 23-mm CB, and 141 PVs 352 times using 28-mm CB. Compared with 28 mm CBs, BFT was shorter and BNT was lower when using 23-mm CBs in all PV locations (all p < 0.001), while BWT was shorter only in superior PVs (see Table 4).

**Table 4:** Parameters of cryo kinetics

PV location	BFT (s)			BNT (ºC)			BWT (s)		
	23-mm CB	28-mm CB	p	23-mm CB	28-mm CB	p	23-mm CB	28-mm CB	p
LSPV	13.7 ± 4.2	23.8 ± 9.1	< 0.001	-52.8 ± 6.5	-46.8 ± 7.1	< 0.001	19.8 ± 7.7	25.3 ± 11.0	0.001
LIPV	14.5 ± 3.4	27.3 ± 7.8	< 0.001	-50.2 ± 4.9	-42.0 ± 4.8	< 0.001	17.5 ± 5.7	17.9 ± 5.9	0.656
RIPV	13.9 ± 4.0	28.1 ± 8.9	< 0.001	-52.6 ± 5.9	-42.3 ± 6.9	< 0.001	20.0 ± 7.6	18.4 ± 8.4	0.237
RSPV	12.1 ± 3.0	21.3 ± 7.6	< 0.001	-56.8 ± 5.1	-49.7 ± 6.7	< 0.001	26.0 ± 6.8	30.4 ± 11.5	0.008
Total	13.6 ± 3.8	25.2 ± 8.8	< 0.001	-53.1 ± 6.1	-45.1 ± 7.1	< 0.001	20.8 ± 7.6	22.7 ± 10.6	0.014

Values are mean ± SD. p: p-value (unpaired Student's t-test). CB: cryoballoon; BFT: balloon freezing time from 0 to -30ºC; BNT: balloon nadir temperature; BWT: balloon warming time from -30 to +15ºC; LSPV: left superior pulmonary vein; LIPV: left inferior pulmonary vein; RIPV: right inferior pulmonary vein; RSPV: right superior pulmonary vein.

Correlation between BNT and BFT (correlation coefficient: 0.77, p < 0.001), and between BNT and BWT (correlation coefficient: - 0.85, p < 0.001) was stronger than that between BFT and BWT (correlation coefficient: - 0.60, p < 0.001) when using 23-mm CB. The same result was found when using 28-mm CB (correlation coefficient: 0.79, - 0.86, and - 0.62, respectively; all p < 0.001).

### PV anatomy and BNT

As mentioned above, D_corrected_ has much stronger correlations with both D_long_ and D_short_, and BNT has much stronger correlations with the two other kinetic parameters as well, D_corrected_ and BNT were chosen as parameters to investigate the relationship between PV diameter and cryo kinetic parameter. To reflect the maximal biological effect and avoid the confounding effect caused by manipulation between different cycles (e.g., occlusion degree, time of freezing circle), the lowest BNT achieved by using the same size of balloon was chosen to analyze each PV.

**Figure 3:**
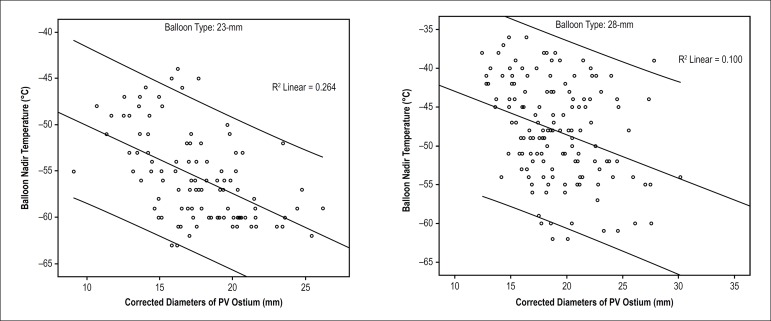
Scatterplot of PV ostium corrected diameters and balloon nadir temperature using two sizes of cryoballoon.

Correlation analysis revealed that the correlation coefficient between D_corrected_ and BNT was -0.51 when ablation was with 23-mm CB, and it was -0.32 with 28-mm CB (both p < 0.001). Correlation between the two parameters was stronger when using 23-mm CB (see Figure 3). However, there was no significant correlation between value of D_short_ / D_long_ and BNT either using 23- (correlation coefficient: -0.11, p = 0.23) or 28-mm CB (correlation coefficient: -0.09, p = 0.30).

**Figure 4:**
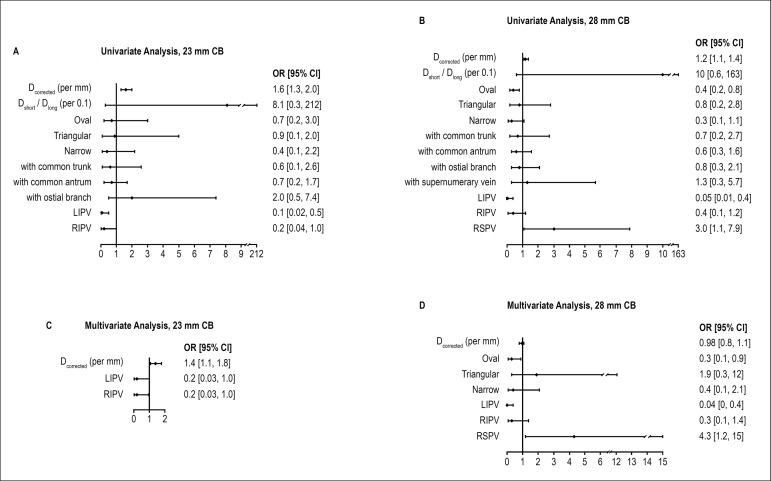
Univariate and multivariate logistic regression analysis for BNT (< -51ºC and ≥ -51ºC). A and C. Univariate and multivariate analysis of 23mm CB. B and D. Univariate and multivariate analysis of 28 mm CB. D_long:_ PV ostium long diameter; D_short:_ PV ostium short diameter; D_corrected:_ Corrected diameter calculated from PV ostium perimeter PV ostium; CB: cryoballoon; LSPV: left superior pulmonary vein; LIPV: left inferior pulmonary vein; RIPV: right inferior pulmonary vein; RSPV: right superior pulmonary vein.

In order to investigate the predict value of PV anatomic parameters for cryo kinetic effect, BNT was transformed into a binary variable with a cut-point of -51ºC (< -51ºC and ≥ -51ºC)^4^ and taken as dependent variable. PV anatomic parameters including D_corrected_, value of D_short_ / D_long_, ostium shape, drainage pattern and location were included in logistic regression model as independent variables. Univariate and multivariate analyses revealed that, among the above-mentioned variables, D_corrected_ [OR, 1.4(95% CI: 1.1 - 1.8), p* *= 0.004] predicted a BNT of < -51ºC when using 23-mm CBs, while an oval shape of PV ostium [OR, 0.3(95% CI: 0.1 - 0.9), p = 0.033] and PV locations [left inferior PV: OR, 0.04(95% CI: 0.004 - 0.4), p = 0.005; right superior PV: OR, 4.3(95% CI: 1.2 - 15), p = 0.025] predicted a BNT of < -51ºC when using 28-mm CB. However, PV drainage patterns did not predict it when using either 23- or 28-mm CBs. (see Figure 4).

## Discussion

### Main findings

This study aimed to investigate the relationship between PV anatomy and cryo kinetics during CB ablation. The present study main findings can be summarized as follows: Firstly, MDCT was accurate and useful in pre-procedural evaluation of PV anatomy for CB ablation of AF; D_corrected_ was a better parameter for ostial measurement than D_long_ and D_short_. Secondly, BNT, BFT and BWT were associated to each other, BNT was a better parameter for evaluating cryo kinetics effect than the latter two. Thirdly, there is an association between D_corrected_ and BNT both when using 23- and 28-mm CBs; D_corrected_ predicted cryo kinetic effect with a BNT of < -51ºC when using 23-mm CB, while PV ostial shape and location predicted the effect when using the 28-mm CB.

### PV anatomy evaluated with MDCT

MDCT images can provide accurate and detailed PVs anatomic information.^10^ Our study found that variations existed in dimensions, ostial shapes and drainage patterns of PVs among different patients and PV locations, which was consistent with prior studies.^11^^,^^13^^,^^14^ Values of PV ostia D_short_ / D_long_ that we studied were between 0.38~1.00, and only 20.8% PVs (50/240) had a round-shape ostia. Therefore, it is a partial evaluation using only D_long_ or D_short_ as PV ostial dimension. Considering PVs compliance and deformation to adapt the CB during procedure, D_corrected_, diameter calculated from the perimeter was more reliable. Correlation analysis on PV ostial dimension measurement also demonstrated D_corrected_ was more representative than the two others.

### CB ablation and cryo kinetics

Cryo kinetics can be evaluated from two aspects: freezing temperature and time course. Furnkranz et al.^4^ found that BNT could predict acute PVI when using 28-mm CB. Ghosh et al.^5^ reported that -30~+15ºC BWT was a strong predictor for pulmonary vein reconnection. The current study revealed that BFT, BNT and BWT had significant correlations to each other, which was higher between BNT and the two others. For this reason, we chose BNT as the representative cryo kinetic parameter for analyzing the relationship between PV anatomy and cryo kinetics. A cut-point of < -51ºC was selected for logistic regression because BNT < -51ºC was invariably associated with PVI, as Ghosh et al.^5^ concluded.

### Relationship between PV Anatomy and Cryo Kinetics

The CB ablation basic technique is to achieve cryoenergy-induced PVI on a condition of appropriate occlusion of PV blood flow and circumferential contact between PV ostia and CB surface, ideally the equatorial region of CB.^15^ Sorgente et al.^6^ found that PV ostium shape was useful in predicting the degree of occlusion. PV ovality^16^ and drainage patterns^9^ were reported to have an impact on AF recurrence in some studies. In this study, though a mild to moderate association was found between BNT and D_corrected_, no association existed either between BNT and PV ostium shape or between BNT and PV drainage pattern. The main reasons for this may be as follows: (1) PV ostia had certain compliance and could deform to adapt to the CB during procedure; (2) Different definitions of PV ostium shape and drainage pattern between studies; (3) cryo kinetic effect is associated with but not equal to occlusion degree or ablation effect.

Compared with 23-mm CB, the association between BNT and D_corrected_ was weaker when using the 28-mm CB. This may be because: (1) 28-mm CB had a higher requirement for PV compliance and “free space” to handle (e.g., PV location, puncturing site of interatrial septum); (2) PV ablated using the 28-mm CB had a smaller ratio of D_corrected_ and diameter of CB in this study, which limited the comparability.

### Efficacy and safety of two CB sizes

Some studies reported that 23-mm CBs was associated with higher success rates but came at the cost of safety, referring mainly to the complication of PN palsy.^15^^,^^17^_,_^18^ PN palsy occurs more frequently in right PVs with an incidence of 2.0% ~ 24.4%.^12^^,^^19^_,_^20^ Our study demonstrated that the overall complication rate were not significantly different between using the two CBs, while ablation using 23-mm CB only had a similar rate of acute PVI on PV level and nonsignificant higher rate on patient level comparing with using 28-mm CB only. It is worth mention that the only one case of PN palsy (1.7%) occurred when using 28-mm balloon, this indicates that, with the improvement of operators’ skills and monitoring methods, smaller CB can be just as safe as the bigger CB while achieving comparable or even higher efficacy when using for the selected patients.

### Study limitation

In this single-center study with a small sample, PV anatomy variations might only partially represent the universal situation among population; BNT cut-point < -51ºC was just used to facilitate the analysis and it is not a cut-point between effective and non-effective ablation, so was the cryo kinetic effect not equal to ablation effect. As SCs were used not only to record PV potentials, but also to support the CBs, real-time PV isolation recording, a more direct and better parameter to evaluate acute ablation effect, could only be achieved in some of the patients. However, this situation is expected to change with the progress of technology and manipulation skills,^21^ and investigation of relationship between PV anatomy and real time isolation will be the future research direction. Current results only apply to the use of first-generation CB. With the spreading use of CB second generation, cryo kinetics needs further discussion. In addition, the evaluation of PV anatomy was carried out with Carto system in electrophysiological lab for convenience and efficiency. Other post processing platforms and reconstructing software could also be used for analysis.

## Conclusions

MDCT images can provide accurate evaluation of PV ostial anatomy and preprocedural guidance for CB ablation. PV anatomy is associated with cryo kinetics, and PV diameter plays a more prominent role when using 23-mm CBs, while PV location is more prominent when using 28-mm CBs.
